# Alternatives to Antibiotics: A Symposium on the Challenges and Solutions for Animal Health and Production

**DOI:** 10.3390/antibiotics10050471

**Published:** 2021-04-21

**Authors:** Todd R. Callaway, Hyun Lillehoj, Rungtip Chuanchuen, Cyril G. Gay

**Affiliations:** 1 Department of Animal and Dairy Science, University of Georgia, Athens, GA 30602, USA; 2 Beltsville Agricultural Research Center, Animal Bioscience and Biotechnology Laboratory, USDA-Agricultural Research Service, Beltsville, MD 20705, USA; hyun.lillehoj@usda.gov; 3 Research Unit in Microbial Food Safety and Antimicrobial Resistance, Department of Veterinary Public Health, Faculty of Veterinary Science, Chulalongkorn University, Bangkok 10330, Thailand; chuanchuen.r@gmail.com; 4 Office of National Programs, USDA-Agricultural Research Service, Beltsville, MD 20705, USA; cyril.gay@usda.gov

**Keywords:** antimicrobials, antibiotic growth promoter, gut health, microbiome, pre/probiotics, antibiotic resistance, phytochemicals, immune modulation

## Abstract

Antibiotics have improved the length and quality of life of people worldwide and have had an immeasurable influence on agricultural animal health and the efficiency of animal production over the last 60 years. The increased affordability of animal protein for a greater proportion of the global population, in which antibiotic use has played a crucial part, has resulted in a substantial improvement in human quality of life. However, these benefits have come with major unintended consequences, including antibiotic resistance. Despite the inherent benefits of restricting antibiotic use in animal production, antibiotics remain essential to ensuring animal health, necessitating the development of novel approaches to replace the prophylactic and growth-promoting benefits of antibiotics. The third International Symposium on “Alternatives to Antibiotics: Challenges and Solutions in Animal Health and Production” in Bangkok, Thailand was organized by the USDA Agricultural Research Service, Faculty of Veterinary Science, Chulalongkorn University and Department of Livestock Development-Thailand Ministry of Agriculture and Cooperative; supported by OIE World Organization for Animal Health; and attended by more than 500 scientists from academia, industry, and government from 32 nations across 6 continents. The focus of the symposium was on ensuring human and animal health, food safety, and improving food animal production efficiency as well as quality. Attendees explored six subject areas in detail through scientific presentations and panel discussions with experts, and the major conclusions were as follows: (1) defining the mechanisms of action of antibiotic alternatives is paramount to enable their effective use, whether they are used for prevention, treatment, or to enhance health and production; (2) there is a need to integrate nutrition, health, and disease research, and host genetics needs to be considered in this regard; (3) a combination of alternatives to antibiotics may need to be considered to achieve optimum health and disease management in different animal production systems; (4) hypothesis-driven field trials with proper controls are needed to validate the safety, efficacy, and return of investment (ROI) of antibiotic alternatives.

## 1. Introduction

Antibiotics have been used widely since the 1950s to improve animal growth efficiency and reduce animal morbidities in animal agriculture [1,2]. The use of antibiotics has reduced the cost of animal-derived protein worldwide, thereby greatly improving the quality of life for billions of people. Although antibiotics clearly control populations of bacteria that are pathogens or opportunistic pathogens, they may also influence animal growth performance through other mechanisms. For example, antibiotics reduce the microbial population of the gut, which results in reduced energy expenditure in the immune system, while some antibiotics have been demonstrated to reduce cytokine production, which is linked with reduced systemic inflammation [3,4,5]. Collectively, the reduction in non-growth energy expenditures by the animal is considered to be responsible for some of the improvements in animal production efficiency. Unfortunately, the use of antibiotics has led to the widespread development and dissemination of antibiotic resistance, which has obviously been a major unintended consequence [6]. However, without antibiotics, enteric pathogenic challenges during the early growth phase of livestock and poultry will cause immense economic losses in the animal meat industry; thus, even with reduced antibiotic use, the benefits of antibiotics must be replicated to ensure food security worldwide. Alternatives to antibiotics are broadly defined as any substance that can serve as a substitute for therapeutic drugs that are increasingly becoming ineffective against pathogenic bacteria, viruses, or parasites.

Due to the global concerns associated with the loss of medically important antibiotics due to regulatory restrictions or the emergence of antimicrobial resistance (AMR), there is a critical need to develop innovative strategies and tools that improve animal health and production efficiency in a fashion similar to how antibiotics were previously utilized, described by Dr. Dixon (National Institute of Allergy and Infectious Disease), Dr. Erlacher-Vindel (World Organization for Animal Health), and Dr. Cyril Gay (Agricultural Research Service; ARS). The United States National Action Plan for Combatting Antimicrobial Resistant Bacteria (CARB) and in nations worldwide was presented by Dr. Jomana Musmar (PACCARB), and this plan discussion touched on the regulatory hurdles and strategic objectives impacting the development of Alternatives to Antibiotics around the world. The third Alternatives-to-Antibiotics Symposium in Bangkok, Thailand addressed the most promising alternatives, including (1) vaccines, (2) microbial-derived products, (3) innovative drugs, chemicals, and enzymes, (4) phytochemicals, (5) immune-related products, and (6) highlighted the regulatory pathways to enable the licensing of alternatives to antibiotics and incentives to support their development.

In general, there is a lack of consensus on what is meant by the term “antibiotic alternatives”. Common antibiotic growth promoters (AGPs) have been routinely used over the last 50 years in modern livestock production without much understanding of their mechanisms of action. However, most of the agents that were discussed under antibiotic alternatives have known mechanisms of action that involve inhibition, alteration, or killing of one or more bacteria. Although the notion that antibiotic alternatives can substitute for the low-level feeding of broad-spectrum antibiotics used to promote growth in livestock is generally acceptable, a clear understanding of the actual effects of antibiotic alternatives is essential. The market for antibiotic alternatives in feed additives is growing rapidly and attracting interest, but the lack of incentives for pharmaceutical companies to invest in their development remains a significant barrier. Therefore, this symposium highlighted promising advances in the research and development of technologies that could potentially replace or decrease conventional antibiotic usage, assessed challenges associated with their commercialization and use, and identified regulatory pathways and incentives to enable their development.

## 2. Individual Seminar Sessions

### 2.1. Session 1: Vaccines: Teaching the Immune System a New Language

The immune system plays a critical role in defending the body, both passively and actively [7,8]. Vaccination derives its name from Jenner’s recognition of differential susceptibility to smallpox in humans based upon previous exposure to a similar pathogen in cattle. This revolutionary concept has improved human health and saved billions of lives worldwide, and has been widely used to improve human health and animal agriculture for the better part of a century. The success of vaccination programs has prevented numerous diseases, thereby reducing the burden of human and animal morbidity and mortality without the need for antibiotic treatment. Although vaccine development is regarded as a mature field that has little new to offer, this is not necessarily accurate and significant challenges remain. However, the need to reduce antimicrobial use has stimulated the development of new exciting vaccine technologies that alert the immune system to threats and may reduce the need for antibiotics that led to the development and maintenance of antibiotic resistance [9].

Vaccines have long been used to generate active immunity against diseases that affect animal health, but their use to prevent diseases that were traditionally controlled with antibiotics has increased recently [9,10]. Additionally, the use of vaccines has resulted in a reduction in the development and selective pressure on antibiotic resistance in humans by preventing viral diseases that are often accompanied by secondary bacterial infections [11]. Vaccines are also increasingly being used to prevent *Salmonella* infection, which is both a food safety issue and a threat to animal health [12,13]. However, despite its importance in enhancing animal health, vaccination is not a “silver bullet” that will eliminate all pathogens, mainly because of the sheer number of pathogens, both established and emerging. For example, the immunization of broilers against necrotic enteritis has encountered significant challenges, described in reports from Dr. Prescott (University of Guelph), Dr. Bielke (The Ohio State University), and Dr. Li (ARS). Filip Van Immerseel (Ghent University) and Dr. Sasai (Osaka Prefecture University), which discussed challenges and opportunities associated with the sheer number and variety of antigens that can elicit an active immune response, and this plethora of antigens can be overwhelming and potentially poses a limit to the total number of specific pathogens that animals can be vaccinated against without producing interference or hypersensitivity.

Broiler chicken production faces many disease problems around the world, and many of these are illnesses that have been traditionally controlled by the use of antimicrobials. Necrotic enteritis is a significant problem affecting poultry health and production efficiency and is caused by *Clostridium perfringens*. Dr. John Prescott suggested that the antibodies may bind to the bacterium itself, preventing replication directly rather than via opsonization. Dr. Lisa Bielke and other researchers described how over 30 peptide antigens from *C*. *perfringens* complicate the search for the best antigen/delivery system, delaying the deployment of a vaccine into production. *Salmonella* vaccination in poultry, swine, and cattle poses an even greater challenge because of the vast number of serotypes and the ability of these serotypes to translocate into the lymph nodes and be incorporated into food products. Dr. Filip Van Immerseel described these challenges along with a discussion of some of the markers of serologic response in food animals.

In this regard, a very exciting development has emerged from a USDA/ARS lab in research from Dr. Crystal Loving, where the Differentiate Infected from Vaccinated Animals (DIVA) or marker vaccine strategy [14] has been used to develop a *Salmonella* vaccine that is active against many swine-associated strains, but also in turkeys. The use of the live attenuated vaccine holds promise for reducing *Salmonella* colonization and carriage in food animals, but it is still associated with risks and barriers to wide acceptance and implementation. Modification of the LPS composition through deletions in *Salmonella typhimurium* did not turn on Toll-like receptor (TLR)-4-mediated inflammation signals, indicating that a specific reaction could target *Salmonella* serotypes such that vaccinated birds could be differentiated from challenged birds (similar to a natural DIVA).

Coccidia are protozoan pathogens in poultry and cattle production, and these food animals are typically fed ionophores to reduce the incidence of coccidiosis. Vaccinations against a broad spectrum of *Eimeria* that target subunits and are designed to be universal against this genus are being developed by researchers at the University of Arkansas and Kut Technical Institute in Iraq led by Dr. Billy Hargis. By reducing the carriage and the incidence of these pathogens, the need to include ionophores in cattle and poultry rations to reduce disease incidence and improve growth efficiency can be reduced, as was demonstrated by Dr. Layton with Vetanco in Buenos Aires. A number of multiple-antigen vaccines against *Staphylococcus* and the coryza (or snot)-causative agent *Avibacterium paragallinarum* were also shown by Dr. Kong of the SEPPIC group to have a direct influence on poultry production efficiency, profitability, and animal health.

Collectively, the results from the first session of the ATA conference demonstrated the broad scope of vaccination solutions for pathogens in food animal production. Nevertheless, numerous challenges associated with the use of vaccines remain unresolved, and there are some unsurmountable obstacles preventing total immunization against all pathogens. However, vaccination remains the most cost-effective and efficacious approach to reduce the loss of production efficiency and animal health resulting from pathogens that impact animal production. By using vaccines to broadly reduce the impact of infectious diseases on production, and in concert incorporate other antibiotic alternative strategies (e.g., phytochemicals, microbial-derived products) with enhanced effects to address the gaps in available intervention used in animal production systems that have traditionally been filled by the broad use of antimicrobials.

### 2.2. Session 2: Microbial-Derived Products

Microbial-derived products originate from various microorganisms (mainly bacteria, yeast and molds) that are productive sources of structurally diverse bioactive metabolites [15] and act as producers of some important antimicrobial drugs and other pharmaceutical products [16]. Recent advances in molecular biological techniques have either improved existing techniques or developed new approaches that have been applied for increasing the availability of novel compounds generated from microorganisms. A variety of bioactive molecules have been launched into markets and become antibiotic alternatives valuable to food animal production. These molecules are now being used for applications other than chemical defenses against infections. During the 3rd Alternatives to Antibiotics (ATA) symposium, microbial-derived products were discussed as approaches to improve animal nutrition, enhance immunity, and increase disease resistance, leading to reduced antimicrobial use in food animal production.

Presentations on advances of next-generation sequencing technology during the symposium highlighted that it is now possible to understand the complexity of microbial communities (microbiomes) in either humans or animals and increase our understanding of the ecology of AMR from a One Health perspective. The human microbiome has been more extensively researched and shown to play an important role in human health [17,18]. Recently, the microbiome of livestock has been sequenced, e.g., cattle [19], pigs [20], and broiler chickens [21]. Using metagenomic sequencing, the latter demonstrated that a full-treatment course of chlortetracycline caused shifts in bacterial abundance and the abundance of resistance genes (resistome) in the structure of the gut microbial ecology of broiler chickens. The changes of resistome were dependent on the specific AMR gene subtypes; for example, the abundance of *tetA* and *tetW* was promoted but multidrug resistance genes (*mdtA*, *mdtC*, *mdtK*, *ompR*, and *tolC*) were inhibited. In addition, the primary host for aminoglycoside resistance genes was shifted from *E. coli* to *Klebsiella* with the therapeutic dose of chlortetracycline [21].

The presentation by Professor Hein Min Tun (University of Hong Kong) was on the use of microbiomes in the gut to address antimicrobial resistance (AMR). The primary strategies included manipulation of the gut microbiome to eliminate antimicrobial resistant bacteria and to increase host immune responses to vaccines. The example was fecal microbiota transplantation (FMT), a microbiome therapy that has been used to effectively treat recurrent *Clostridium difficile* infection [22]. A systemic review showed that 75% of patients who received FMT for recurrent CDI had a resolution of symptoms without recurrence (*n* = 36) [23]. In animals, FMT was successfully used to reduce the development, morbidity, and mortality of porcine circovirus-associated disease in nursery pigs [24] and is under investigation for potential applications on gastrointestinal conditions [24,25]. The role of FMT as an effective approach for reducing intestinal AMR colonization has been suggested [26]. However, this approach is still early in development and the safety, persistence, and efficacy of using FMT for this purpose is a particular concern that must be understood before FMT becomes a viable strategy. The use of alternative microbial-based approaches to manipulating the gut microbiome was amplified by the report of Professor Todd R. Callaway (University of Georgia), whose research focuses on unravelling the microbial ecology of the gut in food animals, and its effects on foodborne pathogenic bacterial populations and AMR transfer [27,28]. The ecology and impact of non-antibiotic approaches to modify the gut microbiome of dairy cattle to enhance milk production; animal health and food safety was emphasized. A large number of approaches have been explored and widely used in the dairy industry, e.g., probiotic and prebiotic feed additives [29], 3-nitrooxypropanol (3-NOP) [30], bacteriophage [31], management practices, dietary changes, organic acid inclusion, and vaccination. Phages isolated from commercial cattle feces are a practical approach to reduce *E. coli* O157:H7 in ruminants as part of an integrated, multi-hurdle system [31]. In addition, utilization of 3-NOP at 40 to 80 mg/kg feed dry matter in dairy cows inhibited methanogenesis, resulting in decreased methane emissions and increased body weight gain without having a negative effect on milk production and composition [30,32].

Probiotics are one of the most common microbial-derived products used in food animal production. The microorganisms most widely used in probiotics are bacteria, of which the most popular used in animal feed belong to the genera of *Lactobacillus*, *Streptococcus*, *Lactococcus*, *Bacillus*, and *Bifidobacterium* [33]. Several *Bacillus* species are formulated in probiotic supplements due to their heat stability and ability to survive the low pH of the gastric stomach [34]. Dr. Park (ARS-USDA) reported the use of a metabolomic approach to explore the impact of using *Bacillus* spp. as a direct fed microbial (DFM) for improving poultry growth performance. Dietary supplementation with *B. subtilis* 747 had profound effects on the levels of a wide variety of chemical metabolites in the chicken gut, and a distinctive biochemical signature was unique to each *B. subtilis*-supplemented group. Chickens fed with a *B. subtilis* 747-supplemented diet showed lower lesion scores of the destructed gut epithelium, significantly decreased the fecal oocyst output, and downregulated expression of proinflammatory cytokines IL-1b, IL-6, IL-2 and INF-g [35]. Hence, the dietary *B. subtilis* supplementation has a potential to replace antibiotic growth promoters in broiler production. In pigs, the stimulatory effect of pig-derived probiotic *L. plantarum* in the modulation of porcine intestinal endogenous host defense peptides (HDPs) synthesis was evaluated [36]. *L. plantarum* strain ZLP001 induced the transcription of jejunal and ileal HDPs genes in weaned piglets with different patterns. The induction seems to be regulated by Toll-like receptor (TLR) 2 recognition, the extracellular signal-regulated kinase (ERK)1/2 and c-jun N-terminal kinase (JNK) signaling pathways, and c-fos and c-jun signaling pathways. These highlight that modulation of endogenous HDPs mediated by *L. plantarum* ZLP001 might be a promising approach to improving intestinal health and enhancing diarrhea resistance in young piglets.

Most probiotics have been granted “Generally Recognized as Safe” (GRAS) status and are considered a good substitute for antibiotics in food animal production [33]. However, feed supplements with microbial-derived ingredients do have a risk of carrying AMR genes that could be transferred to commensal flora and pathogenic bacteria in the gut [37]. A previous study reported that *Lactobacillus* and *Bacillus* isolated from commercial probiotic feed products were found to carry *vanA*. The *vanA*-containing *B. subtilis* additionally harbored *tetW* [37]. This is consistent with the report present by Professor Rungtip Chuanchuen (Chulalongkorn University), demonstrating the presence of *sul1*, *aadA2*, *tetA* and *tetM* in probiotics available for livestock and aquatic animals. An acquired *tetW* flanked by mobile genetic elements was previously observed in commercial strains of *Bifidobacterium animalis* subsp. *Lactis* and lactobacilli used as probiotics or starter culture [34]. Inaccurate label in either numbers or species of bacteria or population level were commonly found among commercial probiotic feed products. These findings highlighted a double-edged sword effect of probiotic usage in livestock applications, and these concerns emphasized the requirement to examine specific probiotic candidate strains to ensure their safety and diminish their potential as contributors to the spread of transferable AMR genes.

### 2.3. Session 3: Innovatives—Drugs, Chemicals, and Enzymes

Antibiotics have had an incredible influence on improving growth efficiency and productivity as well as animal and poultry health for nearly three-quarters of a century [38,39,40], but their benefits must now be replicated by a variety of innovative compounds [41,42,43]. Multiple agents, including non-antibiotic chemicals and enzymes, are being used to replace the beneficial impacts of antibiotics on animal health and production efficiency. Although no single drug or compound can perform all the functions previously performed by antibiotics, the use of a rational mixture of several complementary targeted strategies has been attempted to achieve a synergistic reduction in pathogens and/or improvement of growth efficiency of food animals in the EU project headed by Dr. Baekbo at SEGES in Denmark named “Alternatives to Veterinary Antimicrobials (AVANT)”. Dr. Wang from China Agricultural University discussed replacing the need for the use of antibiotics in production; the use of antimicrobials could be retained for use in ensuring animal health. While it is tempting to think of antibiotic treatments only in terms of their usage in animals, they have also been used to improve the production (and health) of honeybees [44,45]. The need to improve the health of honeybees represents an exciting opportunity for the use of antimicrobial alternatives in very different contexts from those traditionally considered, argued Dr. Judy Chen with USDA-ARS, because the recent loss of honeybees has been linked to pesticides and antimicrobials, making these novel strategies imperative.

During the course of the ATA symposium, several presenters addressed approaches that utilize the activity of the microbial population and their fermentation end products to replace the use of antibiotics as growth promoters and optimize gut function, which was discussed by researchers from DSM in Switzerland and the Philippines. Researchers from Eastman Chemical demonstrated that the addition of specific fermentation end products (e.g., short chain fatty acids (SCFAs)) altered the physiology of the host via changes in energy and nutrient availability, through either production or changes in the intestinal physiology to increase nutrient absorption. For instance, researchers from Naresuan and Kasetsart Universities in Thailand described the influence of fiber addition (e.g., corn stalks, reduced starch) to diets promoting food animal growth was associated with the production of SCFAs and medium-chain fatty acids (MCFAs) by the hind gut microbial population, resulting in increased villus height and tight junction integrity along with the inhibition of bacterial strains including pathogens. These acids subsequently alter the microbiome composition and resultant end products. The effects of dietary ingredients have been demonstrated most profoundly by the use of high-fiber diets (corn stalks) to replace the beneficial effects of tylosin (a macrolide used to prevent liver abscess development) on the incidence of abscesses in feedlot steers in research performed by Elanco Animal Health. In order to replicate the benefits of antimicrobial treatment, it is imperative that we understand the modes of action of antibiotics at the metabolomic, host gene expression, and microbiomic levels in order to understand how the antimicrobials actually confer the animal health and production benefits.

Many methods have been applied to adjust the microbial populations of the gut in humans as well as animals. Prebiotic approaches have become increasingly common, and involve feeding a substrate (e.g., mannanoligosaccharides (MOS) or fructooligosaccharides (FOS)) that can be used by microbes for growth as “colonic food” but is unavailable to the host animal [46,47]. An increased flow of prebiotics as well as other dietary ingredients such as starch to the lower gut, where it can serve as a fermentation substrate for the microbial population, can cause significant alterations in the end products [48,49,50]. End products such as butyrate have been linked with improved gut health and gut integrity [51,52,53]. Many dietary substrates are degraded and fermented by specific members of the microbial population, but some of these organisms may be replaced by the addition of specific enzymes to increase the availability of nutrients. Dr. Poulson from Elanco Animal Health in Poland demonstrated that feeding the carbohydrate-degrading enzyme mannanase to broilers fed mannan resulted in improved gut health measures. Dr. Metta Makhanon described the impact of feeding a complex organic feedstuff (sodium humate) to finishing (fattening) swine, which helped control diarrhea, stimulated the activity of the immune system, and improved the rate of body weight gain. Furthermore, research into the use of organic acids as alternatives to antibiotics across animal and food production has been increasing, and at the ATA conference, combinations of several organic acids were shown by Dr. Yang from the Chinese Academy of Agricultural Sciences to synergistically improve gut integrity and animal performance in swine and broilers. Trihydroxybenzoic acid (THB) was shown by researchers from Kemin Industries to have a significant effect as an anti-coccidial agent against *Eimeria* and reduced bird mortality more than ionophores or other treatments in a challenge model.

Although studies have typically assessed the effects of living microorganisms in the gut, increasing evidence has suggested the importance of bacterial cellular components after they have been lysed and utilized by other microbes. In this regard, research shows that peptidoglycan and lipopolysaccharide from bacterial cell envelopes can have an afterlife in the gut. Peptidoglycan is a rigid structural polymer found outside Gram-positive bacteria and in the periplasmic space of Gram-negative species, which contains some unusual sugars (e.g., N-acetylmuramic acid (NAM)) as well as d-amino acids. Furthermore, lipopolysaccharide (LPS) is a highly active component of bacterial cell envelopes with antigen-recognition sites for the immune system to attack and is involved in colonization of the gut by pathogens such as *Salmonella* [54,55,56]. Changes in the LPS composition have been linked to the development of AMR to specific antibiotics, leading to the exclusion of those antibiotics. LPS has also been linked to reduced integrity of intestinal tight junctions and is associated with “hemorrhagic bowel syndrome” and “leaky gut syndrome”, in addition to facilitating pathogen entry to the host animal [52,57,58]. Little consideration was previously given to the survival of these bacterial cellular components of the gut, but recent research by a team lead by Dr. Nuffenegger from Novozymes and DSM in Denmark has shown that these components can survive for an extended period of time in the gut. Interestingly, specific enzymes and antimicrobial peptides (including chimeric peptides) have demonstrated the ability to kill AMR bacteria and clear the LPS released from bacterial lysis in the gut in research shared by Dr. Wang of the Chinese Academy of Agricultural Science.

Although microbe–microbe interactions within the gut have received much attention, the influence of the host metabolites on the microbial population has not been studied adequately. Researchers from Konkuk University showed that while a high stocking density reduced growth performance in broilers, the addition of gamma aminobutyric acid (GABA) to the diet, which reduces the effects of crowding stress, did not improve growth performance in the absence of antibiotics. Interestingly, DSM researchers demonstrated that vitamin D3 metabolism to 25-Hydroxy-D3 has been shown to play a role in feeding antibiotic-free commercial broilers and could improve their performance.

### 2.4. Session 4: Phytochemicals

Phytochemicals, a term used to describe plant-derived natural bioactive compounds and polyphenols, are the secondary metabolites of plants and their main bioactive compounds and have many proven health benefits [43,59]. Phytochemicals have diverse applications, such as improving nutrient conversion, reducing food spoilage, antimicrobial activity, improving palatability, and enhancing gut health (including immune defense and mucosal growth promotion), making them an ideal category of antibiotic alternatives in the ruminant, swine, and poultry industries [59]. As we enter the post-antibiotic era, plant extracts have shown extremely promising results in terms of zootechnical parameters, immune modulation, and reducing the negative effects of pathogenic challenges, especially with enteric infections. Recent studies have shown that dietary phytochemicals can alter the gut microbiome and modulate mucosal immunity [60,61]. Phytochemicals in general are easy to deliver in animal feed and can be used in solid, dried, and ground forms or as extracts (crude or concentrated), and can also be used as essential oils (EOs; volatile lipophilic substances obtained by cold extraction or steam/alcohol distillation) and oleoresins (extracts derived by non-aqueous solvents), depending on the process used to derive the active ingredients.

In the Scientific Session on phytochemicals, the discussions focused on recent scientific findings, promising novel strategies, and challenges associated with the commercialization of phytochemicals that could serve as alternatives to antibiotics in major agricultural animals (poultry, swine, and ruminants) as well as their modes of action. Although many different phytochemicals have been commercialized globally as feed additives to promote gut health and improve animal productivity, their exact modes of action and potential uses in broader areas of animal health need to be further explored. Furthermore, an industry session on phytochemicals was held to discuss the challenges associated with the commercial application of phytochemicals as feed additives to promote host protective immunity and to decrease the negative effects of enteric diseases in poultry farms where antibiotics are no longer used. The development of a sustainable animal production system using phytochemicals as antibiotic alternatives in the absence of antibiotics will require an enhanced understanding of the modes of action of phytochemicals, including their direct impacts on the microbial population, host immune system, and host animal gene expression.

Dr. J. Furness from the University of Melbourne discussed how micronutrients and phytochemicals are sensed in the gastrointestinal tract, the largest and the most vulnerable body surface that is confronted with a cornucopia of diverse chemicals, pathogens, and physicochemical factors, and the way the host responds to a wide spectrum of sensory signals to optimize nutrition utilization and generate protective responses to these challenges [62]. The gut contains many kinds of specialized receptors, including taste receptors, free fatty acid receptors, and peptide, micronutrient, and phytochemical receptors, many of which are located on enteroendocrine cells and sense nutrients and micronutrients [62]. Receptors in the gut that interact with many phytochemicals commonly belong to the transient receptor potential (TRP) class. However, our knowledge of gut phytochemical receptors in chickens and other food animals is limited, and major challenges remain in determination of the mechanisms of action of phytochemicals, quantitative evaluation of their effects, determination of their interactions with the microbiota, and investigation of their benefits at specific life stages, such as the early life stage and the growing season under environmental threats [59].

With an increasing understanding of the complex intestinal ecosystem and the critical roles of the microbiota in shaping the host immune response as well as gut health [60,61,63], a multifaceted approach using synergistic feed additives may be more effective to reduce the disease effects of complex pathogens such as *Campylobacter*. A multi-pronged approach that combines strategies to reduce *C. jejuni* cecal colonization in poultry gut, inhibit its survival in poultry products, and reduce its environmental persistence during poultry processing by using various phytochemicals was shown to be an effective nonantibiotic method to reduce the risk of human infections [64,65,66]. Trans-cinnamaldehyde (obtained from cinnamon bark), eugenol (from clove oil), and carvacrol (from oil of thyme) all showed effectiveness in reducing *C. jejuni* in the poultry gut and on carcasses as well as in inhibiting *C. jejuni* biofilms on common food processing surfaces [64]. Eugenol was also effective in reducing the survival of *C. jejuni* on chicken skin and wings when applied as an antimicrobial wash or coating treatment [66]. On the other hand, prebiotics such as resistant starches, (e.g., raw potato starch (RPS)), have been shown to support the growth and functions of beneficial members of the intestinal microbiota to increase butyrate production. Butyrate plays an influential role in maintaining colonic homeostasis and moderating host immune responses [67,68]. Pigs fed dietary RPS during the post-weaning period show altered mucosa-associated bacterial communities that are significantly different from those in the untreated group, with reduced populations of proteobacteria, which are commonly associated with intestinal inflammation. The cecum of RPS-fed pigs contains increased levels of butyrate, an SCFA known to affect the host immune status. These positive effects provided protective benefits against *Salmonella* challenge in pigs, indicating the beneficial effects of RPS on the intestinal microbiota, host immune response, and colonic homeostasis to reduce the colonization and shedding of an important human foodborne pathogen [69].

The health benefits of anthocyanin-rich purple potato (PP) in a low-grade inflammation obese mouse model were reported by Zhang et al. from Agriculture and Agri-Food Canada. The gut epithelium acts as a physical and chemical barrier against pathogenic invasion and toxic metabolites, but this barrier function can be compromised by a high-fat diet and gut inflammation. Anthocyanin-rich PP extracts reduced obesity induced by a high-fat diet and ameliorated low-grade gut inflammation through the dose-dependent inhibitory effects of PP on pro-inflammatory mediators such as tumor necrosis factor (TNF)-α, interleukin (IL)-1β, IL-6, and monocyte chemoattractant protein (MCP)-1 and changes in the plasma lipid profile (e.g., total cholesterol, high-density lipoprotein (HDL), and low-density lipoprotein (LDL)). In addition, PP supplementation ameliorated the inflammation-induced loss of tight junction proteins and pro-inflammatory cytokine expression, while restoring the expression of the anti-inflammatory cytokine IL-10 and microbial recognition receptors. These findings demonstrate the role of PP-derived phenolics as promising anti-inflammatory agents that can reduce chronic diseases by promoting gut health.

Tannins are a complex group of polyphenolic compounds found in many plant species that are commonly included in ruminant diets [70]. The use of chestnut and quebracho tannins as phytochemical additives in cattle diets improved feed efficiency by binding dietary proteins, but also affected the gastrointestinal microbiota [71]. The Firmicutes and Bacteroidetes ratio (a parameter loosely associated with energy harvesting) was increased in tannin-supplemented animals, primarily due to increases in *Ruminococcaceae* and decreases in *Prevotella* [71]. Additional studies are necessary to assess the influence of tannins on the rumen microbiota composition and rumen fermentation parameters (e.g., SCFA, methane, ammonia), which affect the energetic status of ruminants.

Traditional Chinese medicine (TCM) often includes phytochemicals as active ingredients, and since the WHO integrated TCM into the 11th ICD (International Classification of Diseases and Related Health Problems), there has been increased interest in adopting TCM approaches into Western medicine. For example, Baitouweng decoction, a traditional Chinese herbal medicine used to treat the diarrhea caused by *E. coli*, was discussed by Dr. H. Dong from Beijing University of Agriculture, China [72]. The Baitouweng decoction described in the Treatise on Febrile Diseases in the Eastern Han Dynasty is a classical TCM prescription that has therapeutic effects on diarrhea and is composed of four herbs: Baitouweng (Pulsatillae Radix), Huang Lian (Coptis Rhizome), Huang Bai (Cortex Phellodendri), and Qin Pi (Cortex Fraxini). Interestingly, despite its good therapeutic effect on *E. coli* diarrhea or its toxins, Baitouweng decoction showed no anti-bacterial activity in in vitro testing. Recent studies have reported that the anti-diarrheal effect was associated with the deactivation of toxins to prevent LPS-mediated damage to the microvascular endothelial cells (MVECs), which form a major defense barrier and help maintain homeostasis in the body.

In the industrial experience session on phytochemicals as antibiotic alternatives, scientists from animal feed companies shared their experimental results on the commercial application of phytochemicals and discussed new challenges associated with the use of phytochemicals to improve gut health in the post-antibiotic era. Dr. Emma Wall from AVT Natural discussed the importance of evidence-based use of plant extracts and how this information is critical to ensure the commercial application of various phytochemicals to improve animal health. Increasing data show that phytochemicals interact with specific receptors in the gut to induce downstream physiological, metabolic, and immune responses that translate into measurable changes in health and performance. Therefore, the application of phytochemicals as feed additives is evolving with time along with changes in poultry production. Next-generation low-dose phytochemicals that target host physiological responses rather than merely exerting a direct antimicrobial effect must be further explored to obtain broader beneficial effects of phytochemicals in commercial animal production.

A thorough understanding of the physiological mechanism triggered by a phytochemical opens avenues to develop multiple-ingredient solutions to improve real-world animal production [59]. Recent advances in “omics” technologies have shown that the gut is an intelligent sensory organ [73] rather than merely a tube for nutrient absorption. Thus, advancements in our understanding of the importance of the gut environment and its barrier function in health will provide a logical approach for the development of effective products that can deliver the benefits of AGPs without increasing the emergence of antibiotic-resistant bacteria. This can be accomplished by using multiple technologies to maintain or strengthen gut barrier function. To this end, scientific principles should be applied to the development of products such that they provide reliable positive benefits to the target animals. Although the animal agricultural feed additive market includes many novel phytochemical-based products, many challenges regarding their application persist, including consistency, safety, and valid scientific proof. This is not surprising because many phytochemical feed additives also modify the microbiota in some way to enrich beneficial bacteria [59,60,61]. Thus, it is likely that a product that can deliver consistent results will need to incorporate two or more components with complementary and/or synergistic mechanisms of action. In addition to understanding the effects on the microbiota, a clear understanding of the effects of the product on the gut epithelial barrier, which is composed of the mucus layer, endothelial cells, and attendant immunological cells and activity, is essential.

### 2.5. Session 5: Immune-Related Products

The immune system is a very powerful, multidimensional tool that can be harnessed to influence animal (and human) health [7]. While vaccination is the best known and accepted approach to modifying and harnessing the immune system, it is not the only route to modulate the immune system to reduce or replace the need for antimicrobial use, and this approach was emphasized by Dr. Heegaard from Technical University of Denmark and Dr. Haagsman from Utrecht University. Unregulated immune response affects the energy and protein stores of animals, and because immune responses are non-growth functions, excess immunostimulation can dramatically affect the efficiency of growth. Non-specific responses such as inflammation can also lead to the development of other conditions that influence animal health and well-being (e.g., autoimmune diseases, leaky gut syndrome). In fact, one of the recognized benefits of antibiotic treatment has been the reduction in systemic inflammation [3,4]. Thus, as Dr. Doug Korver stated, the utilization of alternative approaches that can stimulate the appropriate immune responses while simultaneously minimizing non-specific inflammatory responses is critical.

Selective breeding programs (especially in poultry) have dramatically improved carcass characteristics desired by consumers (e.g., breast size in broilers), but genetic selection focused on this single phenotypic trait has, in some cases, reduced the strength and responsiveness of the immune system [7,74]. Methods that improve the functionality of the innate immune system can be highly influential, conferring upon the host a type of “trained immunity” against a broad spectrum of pathogens [75]. Host defense peptides (HDPs) are antimicrobial peptides (AMPs) with both broad spectrums of activity and sufficient specificity, and can serve as training agents for the innate immune system [43,76,77]. Cathelicidins are HDPs secreted by leukocytes that are active against pathogen invasion in mucosal surfaces [78]. They have been shown to decrease pathogen growth and carriage against subsequent invasion, indicating their role in “training” innate immunity. Researchers from the Vaccine and Infectious Disease Organization demonstrated that innate immune stimulants prevented mortality in chicks caused by yolk sac infections. Another AMP from chickens (NK-lysin) is expressed at a high level in birds infected with *Eimeria*; it has been shown to kill sporozoites via membrane disruption and has also been demonstrated by ARS researchers to work similarly in rainbow trout [78]. High-throughput HDP-screening methods have been developed at Oklahoma State University to allow rapid screening and identification of these compounds for use as antimicrobial alternatives.

Enhancement of the passive immunity of birds and animals via dosing with antibodies from a source other than the host animal’s own immune system is another exciting development. While such stimulation is not new, the utilization of antibodies and immunoglobulins specifically as a replacement for antibiotic treatment is a relatively novel approach that has been tested with IgG and IgY antibodies along with more generalized antibody preparations, as was described by Dr. Lillehoj [79]. Furthermore, special attention was paid during the ATA conference to examining other approaches (e.g., probiotics, phages, and breeding strategies) that stimulate the immune system to recognize and/or act as part of a well-integrated coordinated multiple-hurdle scheme that introduces several sequential interventions that reduce pathogens significantly when applied in series [79]. For instance, the use of egg yolk-derived antibodies specific for enterobactin was shown by University of Tennessee researchers to be effective against Gram-negative pathogens (e.g., *E*. *coli* and *Salmonella*). Elanco Animal Health researchers demonstrated the efficacy of a dried egg product containing anti-IL-10 IgY antibody, which was also demonstrated against the development of necrotic enteritis caused by *Clostridium perfringens* colonization of the gut in broilers.

Although phytonutrient activity and utilization have been discussed previously, the actions of many phytochemicals and microbial-derived products include immunostimulatory properties [41,80,81]. Yeast cell wall products have been previously demonstrated to stimulate immune activity in a variety of food animal species [49,81,82], and were again shown to improve gut health and reduce *S*. *enteritidis* populations in broilers at the ATA conference. This improvement primarily occurred through increased APC, monocyte (and suppressors), and T helper lymphocyte concentrations in the blood, with reduced signs of inflammation in the cecum but not the liver. Moreover, the team from Imunova Analises Biologicas from Curitiba, Brazil, demonstrated that the intestinal integrity was increased in cell wall-treated birds. An ARS research team shared that feeding B-glucans to pigs reduced *Salmonella* shedding, and the gut displayed improved epithelial barrier function (gut integrity) and decreased responses to TLR agonists.

### 2.6. Session 6: Regulatory Pathways to Enable the Licensing of Alternatives to Antibiotics and Incentives for Stakeholders to Support Their Development

As demands for alternatives to antibiotics grow, so will the need to evaluate and regulate these new and upcoming products. Many of these technologies are truly novel and do not fit neatly into any existing product categories. Furthermore, these products must be brought to the marketplace in a rapidly changing scientific and regulatory environment that follows the COVID-19 worldwide pandemic. While the safety and efficacy of alternatives to antibiotics are clearly paramount, consideration must also be given to reducing barriers to market entry for new products; the development of categories and standards for new products and classes of compounds; and the use of “non-standard” approaches such as validated model studies [83,84]. In response to these needs, the U.S. Food and Drug Administration (FDA) has developed a system to facilitate the development of novel alternatives to antibiotics, in which it works in partnership with sponsors to identify pathways for approval early in development.

As a further example, the use of phytochemicals as antibiotic alternatives in agricultural animals is a relatively new field of research, and regulatory positioning and categorization of the key ingredients of phytochemicals is undoubtedly a major challenge for the next decade. Phytonutrients improve the performance of farm animals because they help mitigate the animals’ response to pathogens in their environment through several modes of action [78]. With the increased availability of genomic tools, we will continue to improve our understanding of the modes of action of different plant extracts at the level of genes, receptors, and cell signaling pathways. Food animal production systems face many complicated multi-factorial challenges, many of which involve the interplay of multiple organs, systems, and environmental impacts; such challenges will not be solved with a single silver bullet, but will require integrated approaches that erect multiple hurdles to prevent AMR organism transmission, colonization, and expansion in food animals. Thus, there is a need to provide increased public funding for mechanistic research on phytochemicals to define their efficacy in a consistent manner, thereby preventing false claims while maintaining flexibility in the approval processes for proof of efficacy and safety of products for commercialization.

## 3. Needs and Recommendations from the Panel Discussions

Panel discussions at the end of each session were designed to capture audience perspectives on the problems, needs, solutions, and recommendations to create a roadmap to advance the research and development of novel alternatives to antibiotics. The most promising research would yield alternatives that address all areas related to the reduction in demands for the use of antimicrobials in animal production systems.

## 4. Conclusions

The third International Symposium on Alternatives to Antibiotics: Challenges and Solutions in Animal Health and Production” in Bangkok, Thailand was held to address the following objectives: (1) assess promising research results on alternatives to antibiotics; (2) evaluate the challenges associated with their commercialization and use; and (3) identify the regulatory pathways and incentives for development of these agents. The major conclusions of the discussions can be summarized as follows:

(1) Defining the mechanisms of action of antibiotic alternatives is paramount to enable their effective use, whether they are used for prevention, treatment, or to enhance health and production;
(2) There is a need to integrate nutrition, health, and disease research, and host genetics also must be considered in this regard;
(3) A combination of alternatives to antibiotics may need to be considered to achieve optimum health and disease management in different animal production systems;
(4) Hypothesis-driven field trials with proper controls are needed to validate the safety, efficacy, and return of investment (ROI) of antibiotic alternatives.

Further information and talks presented at two previous meetings and at the third meeting can be found at www.ars.usda.gov/alternativestoantibiotics.
