# Erratum: Callaway et al. Alternatives to Antibiotics: A Symposium on the Challenges and Solutions for Animal Health and Production. *Antibiotics* 2021, *10*, 471

**DOI:** 10.3390/antibiotics10081024

**Published:** 2021-08-23

**Authors:** Todd R. Callaway, Hyun Lillehoj, Rungtip Chuanchuen, Cyril G. Gay

**Affiliations:** 1 Department of Animal and Dairy Science, University of Georgia, Athens, GA 30602, USA; 2 Beltsville Agricultural Research Center, Animal Bioscience and Biotechnology Laboratory, USDA-Agricultural Research Service, Beltsville, MD 20705, USA; hyun.lillehoj@usda.gov; 3 Research Unit in Microbial Food Safety and Antimicrobial Resistance, Department of Veterinary Public Health, Faculty of Veterinary Science, Chulalongkorn University, Bangkok 10330, Thailand; chuanchuen.r@gmail.com; 4 Office of National Programs, USDA-Agricultural Research Service, Beltsville, MD 20705, USA; cyril.gay@usda.gov

The authors would like to make the following corrections to the published paper [1]. There was an error in the original article. Language used by the authors to describe the leadership of an EU project that had been presented at the ATA conference was not clear. The authors would like to delete the sentence “in the EU project headed by Dr. Baekbo at SEGES in Denmark named “Alternatives to Veterinary Antimicrobials (AVANT)” and replace with the sentence “and was described by Dr. Baekbo”.

The correct descriptions of “Section 2.3, Session 3: Innovatives—Drugs, Chemicals, and Enzymes, Paragraph Number 1” are as below:

“Antibiotics have had an incredible influence on improving growth efficiency and productivity as well as animal and poultry health for nearly three-quarters of a century [38–40], but their benefits must now be replicated by a variety of innovative compounds [41–43]. Multiple agents, including non-antibiotic chemicals and enzymes, are being used to replace the beneficial impacts of antibiotics on animal health and production efficiency. Although no single drug or compound can perform all the functions previously performed by antibiotics, the use of a rational mixture of several complementary targeted strategies has been attempted to achieve a synergistic reduction in pathogens and/or improvement in the growth efficiency of food animals and was described by Dr. Baekbo. Dr. Wang from China Agricultural University discussed replacing the need for the use of antibiotics in production; the use of antimicrobials could be retained for use in ensuring animal health. While it is tempting to think of antibiotic treatments only in terms of their usage in animals, they have also been used to improve the production (and health) of honeybees [44,45]. The need to improve the health of honeybees represents an exciting opportunity for the use of antimicrobial alternatives in very different contexts from those traditionally considered, argued Dr. Judy Chen of the USDA-ARS, because the recent loss of honeybees has been linked to pesticides and antimicrobials, making these novel strategies imperative.”

The authors apologize for any inconvenience caused and state that the scientific conclusions are unaffected. The original article has been updated.
